# Multiparametric PET and MRI of myocardial damage after myocardial infarction: correlation of integrin αvβ3 expression and myocardial blood flow

**DOI:** 10.1007/s00259-020-05034-z

**Published:** 2020-09-24

**Authors:** Marcus R. Makowski, Christoph Rischpler, Ullrich Ebersberger, Alexandra Keithahn, Markus Kasel, Ellen Hoffmann, Tienush Rassaf, Horst Kessler, Hans-Jürgen Wester, Stephan G. Nekolla, Markus Schwaiger, Ambros J. Beer

**Affiliations:** 1 Department of Nuclear Medicine, School of Medicine, Technical University of Munich, Munich, Germany; 2 Department of Diagnostic and Interventional Radiology, School of Medicine, Technical University of Munich, Munich, Germany; 3 Clinic for Nuclear Medicine, University Hospital Essen, University of Duisburg-Essen, Hufelandstrasse 55, 45147 Essen, Germany; 4 Department of Cardiology, Klinikum Bogenhausen, Munich, Germany; 5 Department of Cardiology and Vascular Medicine, West German Heart and Vascular Center, University Hospital Essen, University of Duisburg-Essen, Essen, Germany; 6 Department of Chemistry, Institute for Advanced Study and Center of Integrated Protein Science, Technical University of Munich, Garching, Germany; 7 Pharmaceutical Radiochemistry, Technical University of Munich, Klinikum rechts der Isar, Munich, Germany; 8 Department of Nuclear Medicine, University Ulm, Ulm, Germany

**Keywords:** Molecular imaging, PET/CT, Integrin αvβ3, Angiogenesis, Myocardial infarction

## Abstract

**Purpose:**

Increased angiogenesis after myocardial infarction is considered an important favorable prognostic parameter. The αvβ3 integrin is a key mediator of cell-cell and cell-matrix interactions and an important molecular target for imaging of neovasculature and repair processes after MI. Thus, imaging of αvβ3 expression might provide a novel biomarker for assessment of myocardial angiogenesis as a prognostic marker of left ventricular remodeling after MI. Currently, there is limited data available regarding the association of myocardial blood flow and αvβ3 integrin expression after myocardial infarction in humans.

**Methods:**

Twelve patients were examined 31 ± 14 days after MI with PET/CT using [^18^F]Galacto-RGD and [^13^N]NH_3_ and with cardiac MRI including late enhancement on the same day. Normal myocardium (remote) and areas of infarction (lesion) were identified on the [^18^F]Galacto-RGD PET/CT images by correlation with [^13^N]NH_3_ PET and cardiac MRI. Lesion/liver-, lesion/blood-, and lesion/remote ratios were calculated. Blood flow and [^18^F]Galacto-RGD uptake were quantified and correlated for each myocardial segment (AHA 17-segment model).

**Results:**

In 5 patients, increased [^18^F]Galacto-RGD uptake was notable within or adjacent to the infarction areas with a lesion/remote ratio of 46% (26–83%; lesion/blood 1.15 ± 0.06; lesion/liver 0.61 ± 0.18). [^18^F]Galacto-RGD uptake correlated significantly with infarct size (*R* = 0.73; *p* = 0.016). Moreover, it correlated significantly with restricted blood flow for all myocardial segments (*R* = − 0.39; *p* < 0.0001) and even stronger in severely hypoperfused areas (*R* = − 0.75; *p* < 0.0001).

**Conclusion:**

[^18^F]Galacto-RGD PET/CT allows the visualization and quantification of myocardial αvβ3 expression as a key player in angiogenesis in a subset of patients after MI. αvβ3 expression was more pronounced in patients with larger infarcts and was generally more intense but not restricted to areas with more impaired blood flow, proving that tracer uptake was largely independent of unspecific perfusion effects. Based on these promising results, larger prospective studies are warranted to evaluate the potential of αvβ3 imaging for assessment of myocardial angiogenesis and prediction of ventricular remodeling.

## Introduction

Myocardial infarction (MI) and subsequent left ventricular (LV) remodeling is the most frequent underlying cause for the development of chronic heart failure [1, 2]. LV remodeling is characterized by complex cellular and structural processes leading to progressive LV dilatation and deterioration of cardiac function [3]. The extent of myocardial damage is an important determinant of both prognosis and the risk of remodeling after MI [4, 5]. After initial cell death, myocardial tissue in infarcted areas undergoes a healing process associated with inflammation, angiogenesis, fibroblast proliferation, and collagen expression resulting in scar formation [1]. Poor infarct healing and infarct expansion during this phase can influence LV geometry and contribute to progressive remodeling [1, 6]. Modifications of the angiogenic response to ischemia have been investigated as potential treatment strategies to limit infarct size and prevent LV remodeling [7, 8]. The efficacy of these therapies is usually assessed by measurement of perfusion or functional parameters [9, 10]. However, results have been variable and sometimes inconclusive, indicating the need for specific imaging tools for monitoring of angiogenesis and ventricular remodeling. Therefore, novel biomarkers for the assessment of LV remodeling are needed. One of the key players in these processes is the integrin αvβ3. It is a heterodimeric glycoprotein receptor that is highly expressed on endothelial cells during angiogenesis and also on other cells like myofibroblasts [11, 12]. Its expression is upregulated after ischemic myocardial injury in infarcted and border zone regions as part of the early infarct healing process [13–15]. Increased angiogenesis after myocardial infarction is generally considered a favorable prognostic parameter [8, 13, 16–18]. The integrin αvβ3 could therefore represent a novel biomarker after MI to predict adverse LV remodeling processes. Radiolabeled antagonists containing a cyclic Arg-Gly-Asp (RGD) peptide have been used for molecular imaging of αvβ3 integrin expression in oncology and after experimental and human MI using radionuclide imaging [13–15, 17–26]. The first PET tracer used clinically for αvβ3 imaging was [^18^F]Galacto-RGD [27, 28]. This radiotracer is based on the highly αvβ3 specific cyclic pentapeptide cRGDfK, developed by the group of Kessler et al. [29, 30]. There is evidence suggesting that indeed imaging of αvβ3 integrin expression with [^18^F]Galacto-RGD after MI is feasible and associated with post-MI LV remodeling [16]. Imaging of αvβ3 after MI therefore seems promising and αvβ3 could represent a novel imaging biomarker for the assessment of LV remodeling in clinical practice. It has been shown that imaging of αvβ3 expression in patients after MI is feasible using SPECT [12, 13, 31, 32]. Compared with PET, SPECT imaging has several limitations in the clinical setting. Its sensitivity and spatial resolution are lower and quantification of tracer uptake can be challenging. Furthermore, data on αvβ3 imaging after myocardial infarction using PET is limited, and in particular little is known about the relationship of myocardial blood flow and αvβ3 expression in this scenario.

Thus, the purpose of this study was to evaluate the feasibility of [^18^F]Galacto-RGD PET in patients after acute MI for αvβ3 expression assessment. In particular, we examined the correlation of αvβ3 expression with myocardial blood flow and infarct extent as quantified by PET.

## Materials and methods

### Patients

Patient characteristics are summarized in Table 1. Twelve patients, all male, with acute MI were examined with [^18^F]Galacto-RGD PET/CT (mean age 53 ± 12 years, range 35–78 years). Inclusion criteria were history of acute myocardial infarction with successful revascularization within 7 weeks before scanning. Seven (58%) patients suffered a ST-elevation myocardial infarction (STEMI), while 5 patients (42%) suffered a Non-ST-elevation myocardial infarction (NSTEMI). Further inclusion criteria were age over 18 years, and the ability to give written and informed consent. Exclusion criteria were pregnancy, lactation period, and impaired renal function (serum creatinine level > 1.2 mg/dl). The vast majority of patients were not on typical cardiological medication such as ASA, statins, beta blockers, diuretics, or ACE inhibitors/AT II antagonists at the time of the infarction. Only one patient received a drug of the group ACE inhibitors/AT II antagonists due to hypertension. Further patient characteristics including cardiovascular risk factors, number of diseased vessel, and medication taken prior to and after myocardial infarction are summarized in Table 1.

**Table 1:** Patient characteristics and details on infarct characteristics

	**Included patients**	
**Age**	53 ± 12	
**Male**	12 (100%)	
**Cardiovascular risk factors**		
Smoking	7 (58%)	
Hypertension	5 (42%)	
Dyslipidemia	7 (58%)	
Diabetes mellitus	3 (25%)	
Family history	3 (25%)	
**Infarct location**		
Anterior	7 (58%)	
Inferior	3 (25%)	
Lateral	2 (17%)	
**No. of diseased vessels**		
1-vessel disease	8 (67%)	
2-vessel disease	2 (17%)	
3-vessel disease	2 (17%)	
**ECG**		
STEMI	7 (58%)	
NSTEMI	5 (42%)	
**Previous myocardial infarction**	0 (0%)	
**S/p CABG**	0 (0%)	
**Medication**	Prior to infraction	After infraction
ASS	0 (0%)	12 (100%)
Statin	0 (0%)	12 (100%)
Beta blocker	0 (0%)	12 (100%)
Diuretics	0 (0%)	6 (50%)
ACE inhibitors/AT II antagonists	1 (8%)	12 (100%)

All patients underwent PET/CT with [^18^F]Galacto-RGD and [^13^N]NH_3_ within the same imaging session. On the same day cardiac magnetic resonance imaging (MRI) was performed, including CINE sequences for assessment of LV function and volume, as well as late enhancement images after administration of i.v. contrast to evaluate scar formation.

Informed written consent was obtained from all patients. The ethics committee of our university approved the study protocol.

### Radiopharmaceuticals

Synthesis of the precursor and subsequent [^18^F]-labeling of Galacto-RGD and synthesis of [^13^N]NH_3_ were carried out as described previously [33].

### [^18^F]Galacto-RGD PET/CT imaging

Imaging was performed with a Biograph Sensation 16 PET/CT scanner (Siemens, Forchheim, Germany). One hundred twenty minutes after injection of [^18^F]Galacto-RGD (188 ± 19 MBq), an emission scan was performed in the 3D mode covering the area of the heart (three-dimensional mode; 1 bed position, 15 min acquisition time). This time of imaging was chosen to achieve an optimal signal-to-noise ratio based on previous work [28, 34]. Subsequently, an unenhanced low-dose CT scan (120 kV, 25 mAs, collimation 16 × 0.75 mm) was carried out in shallow expiration. For attenuation correction, the CT data were converted from Hounsfield units (HU) to linear attenuation coefficients for 511 keV using a single CT energy scaling method based on a bilinear transformation. Emission data were corrected for randoms, dead time, and attenuation and reconstructed using the ordered-subsets expectation maximization (OSEM) algorithm using 8 iterations and 4 subsets. For noise reduction, a Gaussian filter with a FWHM of 5 mm was applied.

### [^13^N]NH_3_ PET/CT

For perfusion quantification, [^13^N]NH_3_ (740 MBq) was administered. Each acquisition consisted of 21 frames (12 × 10 s, 6 × 30 s, 3 × 300 s). Using an analysis program developed at our institution (MunichHeart/NM), the absolute flow was calculated based on a validated three-compartment tracer kinetic model [35].

### Magnetic resonance imaging

MRI was performed on a 1.5 T imaging system (Achieva; Philips Healthcare, Best, Netherlands) with a dedicated five-channel cardiac coil. After acquisition of scout views, a long-axis view, two- and four-chamber views and short-axis views of the left ventricle were obtained. Gadopentetate dimeglumine (Magnevist; Bayer Schering Pharma AG, Berlin, Germany) was administered at 0.2 mmol per kilogram of body weight via an antecubital venous access. The administration of gadopentetate dimeglumine was followed by a 20-ml saline injection. Delayed enhancement imaging was started 15–20 min after injection of gadopentetate dimeglumine. Optimization of inversion time (TI) was performed using a standard Look–Locker (turbo field echo–echo planar imaging (EPI)) sequence. This pulse sequence is used to determine the correct TI to null the signal intensity (SI) of normal myocardium. Sequence parameters of the Look–Locker sequence include field of view (FOV), 270 × 270 mm; matrix, 128 × 99; slice thickness, 10 mm; in-plane resolution, 2.1 × 2.2 mm; repetition time (TR)/echo time (TE), 40/5.7 ms; flip angle, 15°; and EPI factor 9. The k-space was read out with a centrically reordered technique. All delayed enhancement imaging was performed using prospective ECG-triggering in the breath-hold technique. Data acquisition was performed during the mid-diastole, which was estimated by a time window of minimal cardiac motion. 15–20 min after injection of gadopentetate dimeglumine, a segmented 2D IR–GE (inversion-recovery gradient echo) sequence was started. For both IR sequences, a T1 GE technique was applied. The sequence has the following parameters: TR/TE, 3.6/1.2 ms; bandwidth, 382 Hz per pixel; flip angle: 25°, a typical FOV of 320 × 320 mm (individually adapted); matrix, 160 × 160; and number of signal averages 52. The acquired voxel size was 2.0 × 2.0 × 8 mm, reconstructed as 1.25 × 1.25 × 8 mm.

### Image analysis

**Fig. 1:**
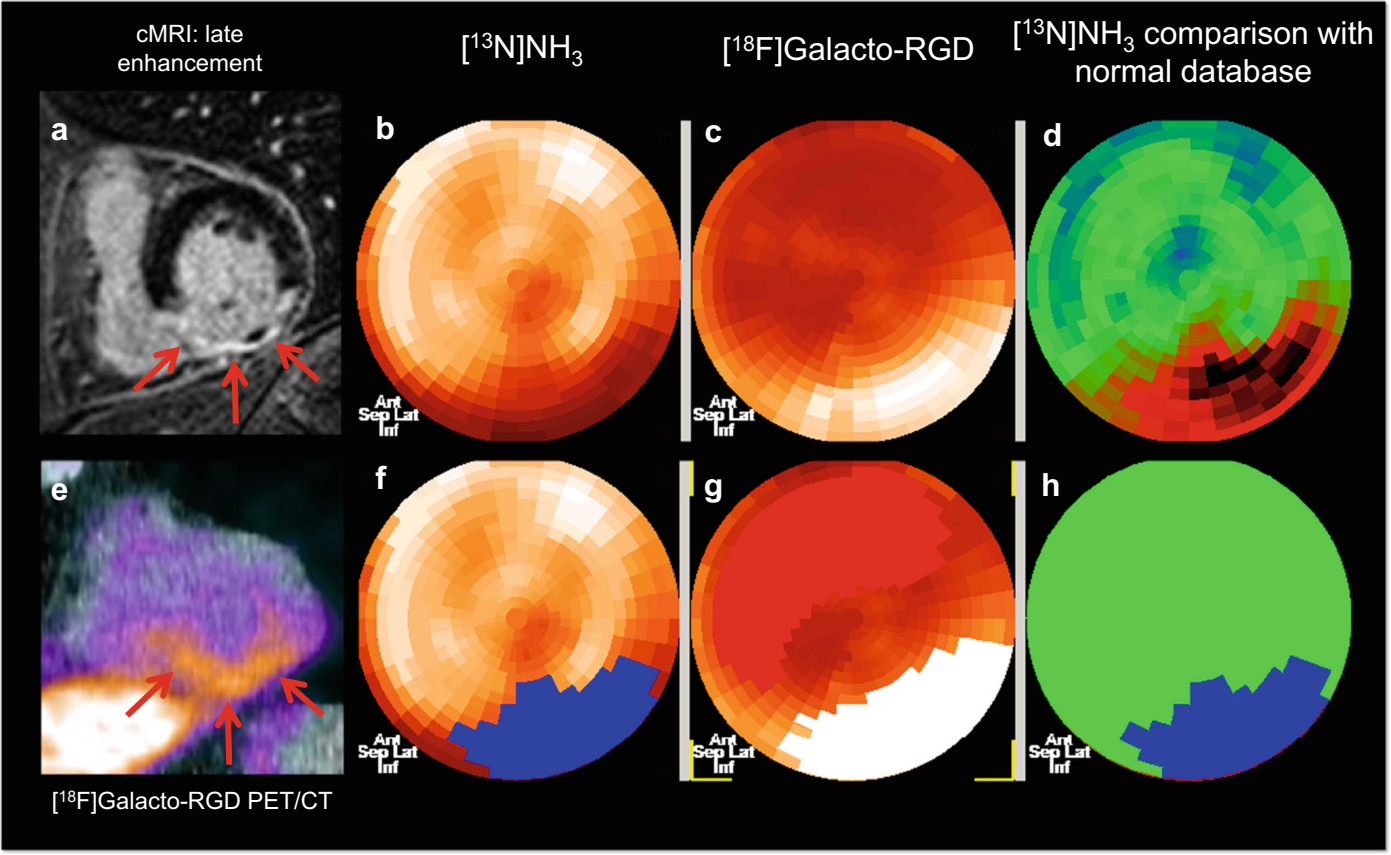
Image analysis in a patient with inferior wall myocardial infarction. Cardiac MRI (A: short axis late enhancement images) shows intense contrast enhancement in the inferior wall with central no reflow phenomenon indicative of a severe transmural infarction (red arrows). [^13^N]NH_3_ PET (B, F: polar maps; D, H: comparison with normal database) shows severely impaired blood flow in the inferior wall, the areas coded in red in D indicate areas with substantially lower flow compared with normal myocardium in a reference population, and the areas in blue in H indicate areas defined as infarcted using a threshold of 0.5 ml/min/g. [^18^F]Galacto-RGD PET/CT (C: polar map without ROIs, G: polar map after manual delineation of the ROI for the infarct area (white) and the remote myocardium without increased [^18^F]Galacto-RGD uptake, with normal perfusion and without late gadolinium enhancement (red); E: short axis PET/CT fusion image) shows pronounced tracer uptake in the area of infarction in the inferior wall with no tracer uptake in normal remote myocardium

The corrected emission scans were calibrated to standardized uptake values (SUVs; measured activity concentration [Bq/ml] × body weight [g]/injected activity [Bq]). Images were analyzed on a multimodality workplace (MMWP) workstation (Siemens, Erlangen, Germany) (see also Fig. 1 for an example of definition of infarct area and area of [^18^F]Galacto-RGD uptake).

For measurement of tracer uptake in [^13^N]NH_3_ PET, the area of infarction was defined by comparison of [^13^N]NH_3_ PET with the normal database.

The ROI for the quantification of the [^18^F]Galacto-RGD uptake was manually drawn into the polar map of the [^18^F]Galacto-RGD PET data. The goal was to reflect the post-ischemic infarct area in this polar map. To achieve this, not only the [^18^F]Galacto-RGD PET itself but also the [^13^N]NH3 PET images and the MRI images were used as reference. In the case of a low [^18^F]Galacto-RGD uptake or an uptake below the remote myocardium, the ROI was based mainly on the [^13^N]NH3 PET and MRI images. To quantify the [^18^F]-Galacto-RGD uptake in the remote myocardium, a ROI map was drawn in the opposite myocardial areas without [^18^F]-Galacto-RGD uptake, which were clearly not affected by the infarction, i.e., in areas without blood flow impairment in [^13^N]NH3 PET or late gadolinium enhancement in cardiac MRI.

Uptake ratios were calculated as follows: (Uptake [Lesion]/Uptake [Remote OR Blood OR Liver], where “Lesion”, “Remote,” “Blood,” and “Liver” refer to the tracer uptake in the infarct area, the healthy remote myocardium, the blood, or the liver, respectively. Lesion/remote ratio was expressed as percentage in order to better illustrate the difference between infarcted and remote myocardium (e.g., a lesion/remote ratio of 20% indicates that the uptake in the infarct area is 20% above the remote myocardium).

Additionally, for all myocardial segments according to the American Heart Association (AHA) 17-segment model, SUVs for [^18^F]Galacto-RGD PET and flow in [^13^N]NH_3_ were calculated and correlated. Definition of infarcted segments was flow of less than 0.5 ml/min/g.

All data sets for the assessment of late enhancement MR imaging of the left and right ventricle were viewed on Totoku monitors (ME 203L, Totoku, Japan). The transmural extent and patterns were evaluated based on the segmentation model of the American Heart Association Guidelines. LE was defined as present only when detectable in two orthogonal planes.

### Statistical analysis

Signal intensities determined for the different regions are expressed as mean ± standard error of the mean (SEM) or in box-and-whisker plots with median, 25th–75th percentile and lowest to highest value. Differences between the different subgroups were evaluated by using a Mann-Whitney test. For comparison of unmatched, continuous variables, the 2-tailed unpaired Student *t* test was used. For linear regression analysis, Spearman’s rank correlation coefficient *r* and the *p* value derived from a two-tailed Student *t*-distribution were computed. Spearman’s correlation coefficient *r* is a measure for the size of the effect. To determine how strong the correlation found is, the classification of Cohen can be used [36]: *r* = .10 corresponds to a weak effect, *r* = .30 corresponds to a medium effect, *r* = .50 corresponds to a strong effect. Statistical significance was assigned for *p* < 0.05. Computations were performed using MedCalc (MedCalc Software, Mariakerke, Belgium).

## Results

### [^18^F]Galacto-RGD uptake after MI

**Fig. 2:**
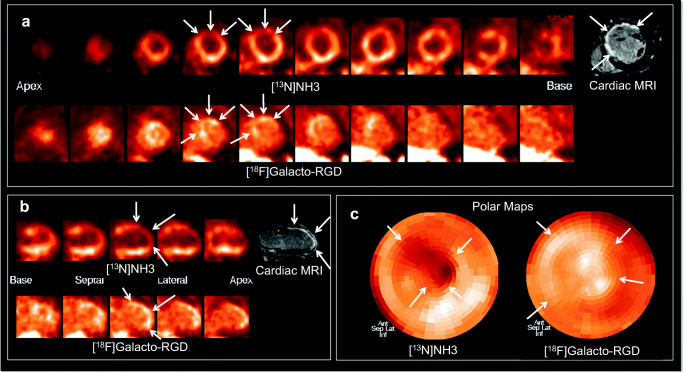
Patterns of [^18^F]Galacto-RGD uptake in myocardial infarction. [^13^N]NH_3_ and [^18^F]Galacto-RGD PET as well as late enhancement cardiac MRI is shown (A: short axis; B: vertical long axis; C: polar maps). A large area of infarction with predominantly transmural contrast enhancement is shown in cardiac MRI in the anterior and anteroseptal wall with severely impaired blood flow in [^13^N]NH_3_ PET (arrows). [^18^F]Galacto-RGD PET shows tracer uptake in the area of infarction; however, note that the tracer uptake also extends to areas adjacent to the infarcted areas into myocardium with normal or only slightly impaired blood flow (arrows), which is especially well demonstrated in the polar maps (C)

**Fig. 3:**
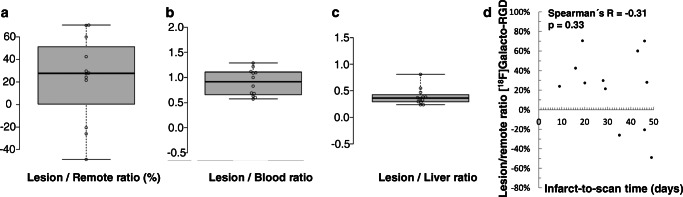
Quantitative analysis of [^18^F]Galacto-RGD uptake. Box-and-whisker plots of [^18^F]Galacto-RGD uptake in infarct areas of all patients, depicted as lesion/remote-ratio (A), lesion/blood-ratio (B), and lesion/liver ratio (C). In 5 patients there was clear tracer uptake visible with a lesion/blood ratio of 1 or more. In the other patients, there was only little tracer uptake, which in 3 patients was even lower than in normal myocardium. Note that lesion/liver ratios were relatively low due to physiologically pronounced liver uptake of [^18^F]Galacto-RGD. There was no correlation between [^18^F]Galacto-RGD lesion/remote-ration and infarct-to-scan time (D)

In vivo PET/CT imaging demonstrated clear [^18^F]Galacto-RGD uptake in 5 (42%) patients within the area of infarction as determined by cardiac MRI and [^13^N]NH_3_ PET (Fig. 1). Note that in some patients, areas of uptake also extended to areas of myocardium adjacent to infarct zones (Fig. 2). The corresponding lesion/remote, lesion/blood, and lesion/liver ratios are reported in Fig. 3. Note also that in some patients, there was even slightly lower uptake in the infarct zone compared with normal myocardium resulting in negative lesion/remote ratios. There was no correlation between lesion/remote ratio and infarct-to-scan time (lesion/remote: *r* = − 0.31, *p* = 0.33, Fig. 3D). As [^18^F]Galacto-RGD shows a relatively high physiological uptake in the liver, lesion/liver ratios were low.

### Correlation of [^18^F]Galacto-RGD uptake with infarct type and infarct size

**Fig. 4:**
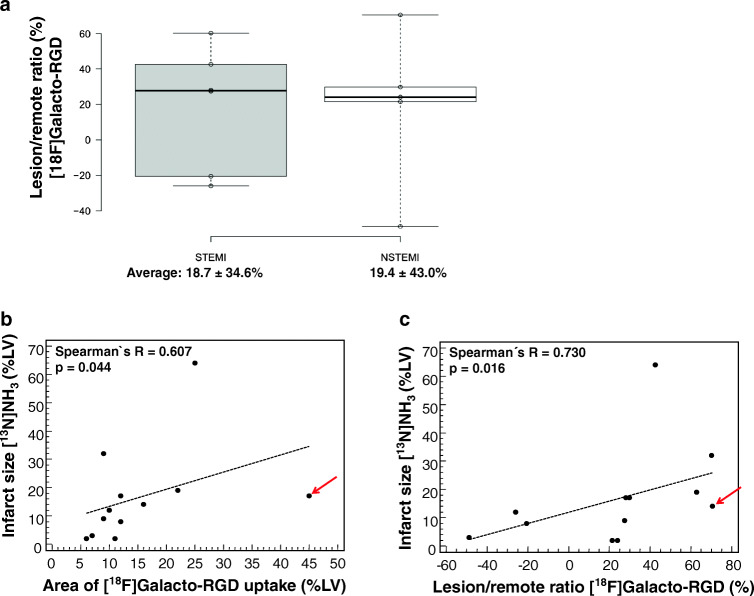
Correlation of [^18^F]Galacto-RGD uptake and infarct type/size. No difference in [^18^F]Galacto-RGD uptake depending on infarct type (STEMI vs. NSTEMI) was observed (A). Also, the correlations of the extent of [^18^F]Galacto-RGD uptake (B) and of the intensity of [^18^F]Galacto-RGD uptake (C) with the infarct area as determined by [^13^N]NH_3_ perfusion PET are displayed. A significant correlation of both area and intensity of [^18^F]Galacto-RGD uptake with infarct size was found. However, the correlation was only moderate with single patients showing intense and/or extensive [^18^F]Galacto-RGD uptake despite a relatively small infarct size (arrow)

The area of [^18^F]Galacto-RGD uptake correlated significantly with the infarct size as determined by [^13^N]NH_3_ perfusion PET. Moreover, the intensity of [^18^F]Galacto-RGD uptake measured as lesion/remote ratio correlated significantly with the infarct size as determined by [^13^N]NH_3_ PET. Note, however, that the correlation was only moderate, as in some patients an intense [^18^F]Galacto-RGD uptake could be seen despite a relatively small infarct zone. No difference regarding [^18^F]Galacto-RGD uptake was observed in STEMI vs. NSTEMI cases (18.7 ± 34.6% vs. 19.4 ± 43.0%, *p* = .97). Results are summarized in Fig. 4.

### Correlation of [^18^F]Galacto-RGD uptake and blood flow

**Fig. 5:**
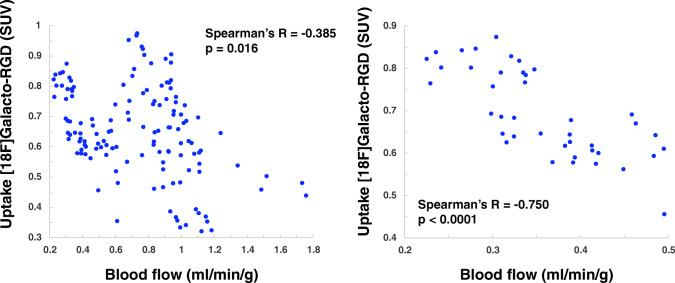
Correlation of [^18^F]Galacto-RGD uptake and myocardial blood flow. Results are shown for all myocardial segments (17-segment model; A) and for the infarcted segments only (B, threshold below 0.5 ml/min/g). A significant but moderate inverse correlation of [^18^F]Galacto-RGD uptake and blood flow for all segments was measured. Note, however, that some segments showed intense [^18^F]Galacto-RGD uptake but only slightly or no impaired blood flow. This corresponds to the visual analysis where [^18^F]Galacto-RGD uptake was seen not only within but also adjacent to areas of infarction in the border zone. When only looking at segments with infarction (B), the inverse correlation was more pronounced, which shows that uptake of [^18^F]Galacto-RGD was not dominated by unspecific perfusion effects

Based on the AHA 17-segment model, tracer uptake of [^18^F]Galacto-RGD and blood flow, as measured by [^13^N]NH_3_, were correlated for each segment. A weak to moderate, but significant, inverse correlation of blood flow and [^18^F]Galacto-RGD uptake for all segments was measured (Fig. 5A). Note that areas of high [^18^F]Galacto-RGD uptake were also seen in areas with relatively normal or only slightly impaired blood flow as well. This is in line with the visual observation mentioned above, confirming that [^18^F]Galacto-RGD uptake could be measured not only within infarcted areas but also adjacent to infarcted areas in the border zone.

In a subgroup analysis, only areas of infarcted myocardium using a threshold of blood flow less than 0.5 ml/min/g were evaluated. In these areas, a substantially stronger and highly significant inverse correlation of blood flow and [^18^F]Galacto-RGD uptake (Fig. 5B) was found. This shows that [^18^F]Galacto-RGD uptake is not substantially biased by unspecific perfusion effects, as good uptake was notable also in segments with very little perfusion.

## Discussion

This is the first study investigating the association between myocardial blood flow and αvβ3 integrin expression in patients after MI using [^18^F]Galacto-RGD and [^13^N]NH_3_ PET, respectively. [^18^F]Galacto-RGD uptake in and adjacent to areas of MI could be visualized and quantified in a subset of patients and significantly correlated with infarct size and impairment of myocardial blood flow. Thus, PET/CT imaging of αvβ3 integrin expression could represent a novel promising imaging approach for the evaluation of myocardial angiogenesis, which is potentially affecting left ventricular remodeling.

### Patterns and intensity of [^18^F]Galacto-RGD uptake

While [^18^F]Galacto-RGD has already extensively been evaluated for imaging αvβ3 expression in malignant lesions, studies with [^18^F]Galacto-RGD and PET also indicated sufficient signal intensity for visualizing increased αvβ3 integrin expression in benign lesions such as chronic skin inflammation and infarcted myocardium [20, 37]. Up to now, data on imaging of αvβ3 after myocardial infarction is limited [18, 23].

This study demonstrates that αvβ3 expression can be visualized and quantified by [^18^F]Galacto-RGD PET in or adjacent to areas of myocardial infarction in humans. Previous preclinical data in a rat model of ischemia-reperfusion strongly suggest that the signal from [^18^F]Galacto-RGD is specific for αvβ3 expression and correlated with myocardial angiogenesis on a immunohistochemical level [20]. Moreover, it has been shown that [^18^F]Galacto-RGD has a high affinity and selectivity for the α_v_β_3_ integrin in vitro as well as receptor-type specific accumulation in vivo in αvβ3 integrin positive tumors preclinically and clinically [19, 27]. In summary, these data suggest that the PET signal measured with [^18^F]Galacto-RGD in our study represents myocardial αvβ3 expression. However, from the PET signal alone, it cannot be differentiated whether the signal derives predominantly from αvβ3 expressed on endothelial cells or from other structures involved in myocardial repair processes after acute MI including macrophages or myocardial myofibroblasts. One study using the integrin αvβ3/αvβ5 specific SPECT tracer RIP has reported a predominant association of tracer uptake and myocardial myofibroblasts [38]. However, in a study using [^18^F]Galacto-RGD in a rat model of ischemia-reperfusion, tracer uptake was predominantly associated with endothelial cells and therefore myocardial angiogenesis [14]. In another study by Dobrucki et al., it has been demonstrated that angiogenesis in a rat model of infarction may not only be visualized by performing SPECT after the administration of a [^99m^Tc]-labeled chelate-peptide conjugate containing an RGD motif; it has also been shown that the angiogenic response may be altered by the application of insulin-like growth factor-1 and then monitored using this imaging approach [31].

Furthermore, we observed an increased [^18^F]Galacto-RGD uptake both in the center of the infarct and in the border zone. It has already been shown that there is an increased neoangiogenesis in the border zone of the infarct, which is associated with an increased αvβ3 expression. This has been demonstrated in a study on a rat infarct model, where an increased CD31 expression in the infarct periphery was found together with an increased uptake of a [^68^Ga]-labeled RGD PET tracer as a sign of increased αvβ3 expression 4 weeks after infarct induction [21]. Furthermore, it has recently been shown that increased neoangiogenesis also occurs in the infarct center through the formation of new vessels sprouting from the endocardium [39].

These preclinical data support that also in the clinical setting, targeting of αvβ3 expression by [^18^F]Galacto-RGD might be an interesting surrogate parameter of ventricular angiogenesis and subsequent remodeling and could also be used for the evaluation or monitoring of new therapeutic approaches.

### Correlation of [^18^F]Galacto-RGD uptake, blood flow, and infarct size

The relationship between αvβ3 expression and myocardial perfusion after myocardial infarction has not yet been sufficiently investigated. In this study, the tracer uptake of [^18^F]Galacto-RGD correlated moderately with infarct size and was more pronounced in areas with more restricted blood flow as quantified by [^13^N]NH_3_ PET. Although the correlations found are only moderate, it should be noted that it was determined using Spearman’s rank correlation coefficient, a test that is insensitive to outliers, and that a strong effect of this correlation can be assumed, which means that the correlation is highly likely to be relevant [36]. Therefore, these data suggest that the severity of MI is interconnected to the intensity of integrin αvβ3 expression about 3–4 weeks after MI. Moreover, [^18^F]Galacto-RGD uptake could also be seen adjacent to the infarcted areas in myocardial segments with no or only little impairment of blood flow. This suggests that myocardial αvβ3 expression is not limited to the infarct zone itself but can also be found adjacent to it in the border zone. This supports the hypothesis that tracer uptake correlates with myocardial angiogenesis, which should be most pronounced in the border zone.

The finding that [^18^F]Galacto-RGD uptake was even stronger inversely correlated to blood flow within the infarct zones themselves indicates that tracer uptake is not dominated by unspecific perfusion effects and strongly suggests specific tracer uptake. There is first evidence that increased αvβ3 expression after MI is a favorable prognostic factor regarding LV remodeling [18], which we could not show in this feasibility study due to the small sample size.

We observed that the mean uptake of [^18^F]Galacto-RGD was several times lower than reported for αvβ3 integrin expressing tumors [28, 40, 41]. This can on the one hand be explained by biological phenomena, as preclinical data suggest that [^18^F]Galacto-RGD predominantly binds to endothelial cells after MI, which are of relatively low density, when compared with a tumor with integrin expression both on endothelial and tumor cells. On the other hand, physical factors might play a role as the volume of MI zones was smaller compared with most tumor lesions in the reported studies, which usually were larger than 20 mm in diameter. As the spatial resolution of PET is limited, spill-out to adjacent tissue with low tracer uptake artificially reduces the measured uptake in lesions smaller than ~ 20–25 mm, the so-called partial-volume effect. To improve uptake and target-to-background ratios, a potential solution might be tracer optimization by multimerization, which has been shown to increase signal intensity from αvβ3 integrin expressing tissues [42, 43]. However, a comparison of various monomeric and multimeric integrin αvβ3 imaging tracers from our group showed no significant differences concerning image contrast in a preclinical model of myocardial infarction [22].

### Comparison to existing studies in humans

To date, there are only a limited number of studies on PET imaging of the αvβ3 integrin after myocardial infarction. In one study 27 patients (21 patients after STEMI and 7 patients with chronic coronary artery occlusion (CTO)) and 9 healthy subjects were examined using [^18^F]-fluciclatide (another αvβ3 targeting tracer) PET/CT and MRI [18]. In line with our study, it was demonstrated that there is an increased αvβ3 integrin expression in the infarct area. Interestingly, an increased αvβ3 expression was associated with an improvement of wall motion. It was also shown that in patients with CTO no uptake was visible in old infarction scars and in healthy volunteers no myocardial uptake was detectable. A major difference to our study is that no information on perfusion was provided. Accordingly, the findings that integrin expression correlated inversely with blood flow and that integrin expression was elevated in the perfused border zone are particularly new. In another study, 23 patients after myocardial infarction were examined with [^68^Ga]-PRGD2, another αvβ3 integrin tracer, using PET/CT [23]. Similar to our study, an increased αvβ3 expression was found in the infarct area and integrin expression was elevated in the patient population up to 2.5 months after infarction.

### Comparison to other imaging modalities

Concerning alternative modalities for molecular imaging of αvβ3 expression, SPECT imaging has already been used clinically in patients after MI, however has a lower sensitivity and limited resolution [32]. Concerning MRI, several preclinical studies reported the successful imaging of αvβ3 integrin expression in atherosclerosis [44, 45]. The higher resolution of MRI combined with excellent soft tissue contrast surely is an advantage compared with PET. However, in MRI significantly higher amounts of contrast agents and probes have to be used compared with PET, which requires only the administration of probes in the microgram range. Therefore, side effects of most PET tracers are limited or not present at all. This facilitates translation of results of PET tracers into the clinical setting, as demonstrated for [^18^F]Galacto-RGD.

### Study limitations

The main limitation of our study is the small patient population. However, this study was primarily focused on the aim to investigate the association of myocardial blood flow with αvβ3 expression in patients after MI and we were the first to describe the inverse relationship. Another general limitation is that the radiotracer [^18^F]Galacto-RGD exhibits a relatively high liver uptake. This may complicate the assessment of integrin expression in case of inferior wall infarctions. However, it has to be stressed that this uptake has to be classified as specific, because the comparison of different αvβ3-targeting tracers with different chemical structure, size, and also polarity showed that they all had an intensive liver uptake, which could be blocked effectively [42]. Finally, the variable timepoint of scanning after MI represents a limitation, which was mainly due to logistic reasons, as depending on the general state of the patients, the quite extensive imaging protocol could not be performed in all patients to the exactly same time after MI. On the other hand, in our patient cohort, which is concordant with the other αvβ3 integrin PET imaging studies in patients after myocardial infarction [18, 23], there was no correlation between the time period of image acquisition after MI and [^18^F]Galacto-RGD uptake, so that timing seems to have only a minor impact at least in the first couple of weeks after infarction.

## Conclusion

We demonstrate that [^18^F]Galacto-RGD PET/CT allows the visualization and quantification of integrin αvβ3 expression in a subset of patients after MI and that αvβ3 expression is inversely correlated with myocardial blood flow in this scenario. Based on these promising results, further prospective studies are justified and warranted to test the clinical value of PET-based αvβ3 expression assessment as a prognostic marker for left ventricular remodeling and prognosis after MI. Combining the high sensitivity of PET with the excellent anatomical detail provided by CT or MRI in hybrid systems, this approach has the potential to map specific molecular signals of the myocardium with high accuracy [46].

## Abbreviations

PET: Positron emission tomography
SPECT: Single photon emission computed tomography
RGD: Arginine glycine aspartate
MI: Myocardial infarction
MRI: Magnetic resonance imaging
CT: Computed tomography
3D: 3 dimensional
SUV: Standardized uptake value
ROI: Region of interest
TB: Target-to-background
SEM: Standard error of the mean
